# The Potential of Deep Learning in Underwater Wireless Sensor Networks and Noise Canceling for the Effective Monitoring of Aquatic Life

**DOI:** 10.3390/s24186102

**Published:** 2024-09-20

**Authors:** Walaa M. Elsayed, Maazen Alsabaan, Mohamed I. Ibrahem, Engy El-Shafeiy

**Affiliations:** 1 Department of Information Technology, Faculty of Computers and Informatics, Damanhour University, Damanhour 22511, Egypt; 2 Department of Computer Engineering, College of Computer and Information Sciences, King Saud University, Riyadh 11543, Saudi Arabia; malsabaan@ksu.edu.sa; 3 School of Computer and Cyber Sciences, Augusta University, Augusta, GA 30912, USA; mibrahem@augusta.edu; 4 Department of Electrical Engineering, Faculty of Engineering at Shoubra, Benha University, Cairo 11672, Egypt; 5 Department of Computer Science, Faculty of Computers and Artificial Intelligence, University of Sadat City, Sadat City 32897, Monufia, Egypt; engy.elshafeiy@fcai.usc.edu.eg

**Keywords:** adaptive integrated filters, deep learning, noise cancellation, UWSNs, monitor aquatic life

## Abstract

This paper describes a revolutionary design paradigm for monitoring aquatic life. This unique methodology addresses issues such as limited memory, insufficient bandwidth, and excessive noise levels by combining two approaches to create a comprehensive predictive filtration system, as well as multiple-transfer route analysis. This work focuses on proposing a novel filtration learning approach for underwater sensor nodes. This model was created by merging two adaptive filters, the finite impulse response (FIR) and the adaptive line enhancer (ALE). The FIR integrated filter eliminates unwanted noise from the signal by obtaining a linear response phase and passes the signal without distortion. The goal of the ALE filter is to properly separate the noise signal from the measured signal, resulting in the signal of interest. The cluster head level filters are the adaptive cuckoo filter (ACF) and the Kalman filter. The ACF assesses whether an emitter node is part of a set or not. The Kalman filter improves the estimation of state values for a dynamic underwater sensor networking system. It uses distributed learning long short-term memory (LSTM-CNN) technology to ensure that the anticipated value of the square of the gap between the prediction and the correct state is the smallest possible. Compared to prior methods, our suggested deep filtering–learning model achieved 98.5% of the sensory filtration method in the majority of the obtained data and close to 99.1% of an adaptive prediction method, while also consuming little energy during lengthy monitoring.

## 1. Introduction

Wireless sensor networks (WSNs) are the most powerful and influential technology of the twenty-first century. They do not require any special infrastructure or monitoring and, therefore, open up a new avenue for use in a variety of applications [1]. Sensor networks comprise thousands of low-power micro-sensors whose primary purpose is to detect and report specific events to a base station. Due to limited battery power, these nodes have minimal memory and processing capabilities. WSNs are simple to set up, but they require that data arrive at their destination on time. The most important aspect of the wireless sensor network’s life is its energy consumption, which is limited by the sensor node’s small battery capacity. A sensor node’s three primary functions are sending, receiving, and sensing. Data transmission is the most energy-intensive function. As a result, if we wish to extend the network’s life, efficiency-raising and data-filtering procedures will be required. Because of the rapid growth of communication and information equipment and technologies in recent years, data traffic movement over networks has increased. Current simple network control systems, however, are considered to be incapable of dealing with such a massive rise in traffic. Deep learning, which has made significant advances in research, is a technology that enables network managers to achieve new intelligent traffic control. Deep learning has shown effectiveness in several fields of information science, including image recognition, speech recognition, robotics, self-driving, and natural language processing. Additionally, extensive training in network traffic monitoring has begun, and additional progress is predicted [2].

Underwater wireless sensor networks (UWSNs) are gaining popularity as a means of monitoring and managing aquatic life. They are, nevertheless, among the most failure-prone branches of Wireless Sensor Networks. UWSNs confront numerous obstacles, including limited memory and bandwidth, high propagation, route loss at higher levels, and high noise volume. Another component influencing acoustic channel performance is underwater noise. As a result, deep learning technology is utilized in conjunction with computational intelligence (CI) techniques to identify undiscovered resources in the water. The capacity of a system to accomplish a certain task, such as data integrity or pure/free-noise and intact experimental monitoring below the sea, is referred to as CI. Currently, data are seen as the essence of all entities in nature, encompassing humans, robots, and devices, such as those inside the Internet of Things (IoT). As a result, the obtained data must be correct, complete, and meet the needs of a certain assignment [3].

## 2. Motivation and Contribution

The efforts of this work were devoted mainly to monitoring aquatic life and overcoming communication issues, such as restricted energy, limited memory, loss of data, and noisy readings through distributed UWSNs. Therefore, the contribution of this paper is aimed at solving problems of communication data-induced sensors dispersed in UWSNs, by proposing a new architecture for the distributed implementation of deep learning in an underwater wireless sensor network. The distributed application distributes the deep learning middle layer into each sensor to reduce traffic and noise as well as enhance the data processing speed and lower energy usage. This is accomplished by lowering the number of connections, condensing the multi-path handover, and filtering the data delivered. The predictive filtration learning method was performed in two steps: First, two adaptive filters, the finite impulse response (FIR) filter and the adaptive line enhancer (ALE), were integrated into the underwater sensor nodes. The integrated filter is proposed to remove undesirable noise from the signal by achieving a linear response phase and passing a signal without distortion using FIR filters. Further, the ALE filter perfectly isolates the noise signal from the measured signal to retrieve the signal of interest. Second, the two filters were embedded at the cluster head (CH) level. The adaptive cuckoo filter (ACF) evaluates if an element is a member of a set through positive impulse matches; if it is conceivable, it is “possibly in a set”; otherwise, false negative impulses are “definitely not in the set”. We also utilized the Kalman filter to calculate the state values of the dynamic underwater sensor networking system in such a way that the expected value of the square of the difference between the forecast and the correct state is as little as possible. Long short-term memory (LSTM) technology is also used in the proposed approach to developing computational intelligence (CI) strategies for anticipating new resources and reducing noise-accompanied data in the water.

## 3. Related Works

Generally, machine learning (ML) is defined by sensor network designers as a set of tools and techniques used to build prediction models. Experts in machine learning, however, acknowledge that it is a rich topic with a wide range of themes and patterns. In the year 2020, Gupta et al. published a survey that used deep learning approaches to determine the untapped resources contained in water. Gupta referred to a system’s ability to acquire a certain task from data or experimental surveillance beneath the water. The data gathered should be correct, complete, and meet the criteria of the task at hand. Underwater data gathering is difficult, due to sensor mobility caused by sequences of water drifts per second. The authors discovered that a significant amount of packet drop occurs as a result of underwater conditions that impede the data collection procedure. Furthermore, different systems for collecting data below the water’s surface already exist, but they are not properly organized [4].

In January 2021, Rani et al. introduced a chapter for readers regarding the concept of sensor networks in underwater environments. The scientists also discussed the potential ways for recognizing underwater activities using a sensor that evaluates real-time information. The authors demonstrated that there are a few problems connected with sensor networks. As a result of their feasibility and adaptability in complicated problem contexts, machine learning (ML) approaches are perfect for successfully resolving such difficulties. Therefore, numerous ML strategies have been described to improve the operational performance of WSNs, particularly underwater WSNs (UWSNs). The goal of this work is to comprehend the ideas of UWSNs and the role of ML in addressing UWSN performance difficulties [5].

In the year 2022, Prajapati and Joshi developed a cluster head election method that circulates the cluster head role among nodes with higher energy levels than the remaining nodes in the network. The researchers used a combination of LEACH and deep learning to extend the network life of the WSN. A convolutional neural network (CNN) was used to select cluster heads in this proposed method. In this study, they combined the benefits of a deep learning method with LEACH to improve network performance. In comparison to the usual LEACH process, the simulation results clearly show that the proposed methodology performs better. Deep learning is used to select the CH, which expands the WSN’s network longevity [6].

In May 2023, Uyan et al. developed machine learning (ML) models to forecast network characteristics and energy consumption of underwater nodes as supplemental methods to optimization models. To that purpose, the authors used Scikit-learn (1.5.2) and Keras (2.12.0) are powerful tools for building and deploying ML models, for creating several regression models and neural network-based models and then examined their performance using score and error measures. They attempted to run their optimization model with various combinations to acquire data for the models. In addition, they have advocated packet duplication and multi-path routing methods in the literature to meet communication dependability criteria. Furthermore, underwater sensors may transmit sensitive data that must be hidden to prevent eavesdropping. The most generally used method for improving network security is cryptographic encryption. Nonetheless, data encryption necessitates computations to cypher the data, which uses extra energy and reduces the network’s lifespan. To address these issues, an optimization model has been suggested to assess the effects of multi-path routing, packet duplication, encryption, and data fragmentation on the lifetime of UASNs. However, the proposed optimization model’s solution time is extremely long, and it does not always produce viable solutions. To that purpose, different regression and neural network methods were proposed in this study as supplemental methods for optimization models for predicting the network parameters and energy consumption of underwater nodes [7]. In the same year, Sathish et al. investigated distance-dependent RSSI localization techniques. The placements of the subsea nodes were determined by the authors, and the MEEs can be approximated. A network field of one hundred meters on each side and one hundred meters in total length was required to take distance measurements. The sensor nodes were given the freedom to communicate with one another in both directions. The living nodes in the network, known as the securing nodes, were the only nodes in the network that did not move from their initial position at any point in time. To acquire MEE readings, the sensor node was distributed at random, and the process took place while the readings were being collected. Following the arbitrary positioning of the sensor nodes, a series of experiments were carried out [8].

In October 2023, Islam et al. attempted to monitor underwater sensors, which may carry critical data that must be concealed to avoid eavesdropping. To address these issues, an optimization model is developed to assess the impact of multi-path routing, packet duplication, encryption, and data fragmentation on the lifetime of UASN. However, the proposed optimization model has a very long solution time, and there are occasionally no feasible solutions available. As a complement to optimization models, this paper proposes various regression and neural network methods for predicting the network characteristics and energy consumption of underwater nodes [9]. El-Shafeiy et al. introduced the MCN-LSTM technique, which leverages deep learning by combining Multiple Convolutional Networks and long short-term memory networks. This combination of techniques detects anomalies in multivariate time series data, allowing for the identification and flagging of unexpected patterns or values that could indicate water quality issues. Water quality data discrepancies can have far-reaching consequences. A method for detecting anomalies in water quality data in real time was presented, using Multiple Convolutional Networks and long short-term memory (MCN-LSTM) technology. The quantitative results demonstrate MCN-LSTM’s potential to improve decision-making processes while avoiding negative consequences from undetected irregularities [10]. The same year, Ali and Jayakody introduced the SIMO-Underwater Visible Light Communication (UVLC) technology. It is a sort of underwater communication technology that transmits data through visible light, specifically the blue-green spectrum. This system used a Single Input Multiple Output (SIMO) design, which means it had one transmitter and several receivers to improve communication reliability and performance. UVLC systems obtained higher data rates than standard acoustic communication systems, making them appropriate for applications that require large bandwidths. In addition, the blue-green spectrum was chosen due to its lesser absorption in water, allowing for more effective transmission. However, the study encountered significant problems, including absorption, scattering, and turbulence in water, which reduced signal quality and range [11]. Following, Ali et al. reported a performance evaluation of vertical Visible Light Communication (VLC) links in mixed water media, which is an exciting field of research, particularly for underwater communication systems. This study typically involved examining several performance indicators such as the bit error rate (BER). As well as an outage probability under varying situations like high turbulence, pointing errors, and transceiver misalignment [12].

In February 2024, Zhang et al. investigated intelligent optimization algorithms, and robot collaboration is an intriguing and complicated topic. They classified optimization approaches in aquatic monitoring mediums as follows: (1) Intelligent Optimization Algorithms: these algorithms, such as the Chemical Reaction Optimization (CRO) algorithm, combine elements from genetic algorithms, simulated annealing, and ant colony optimization to determine the best node deployment strategies. (2) Robot Collaboration: Robots are employed to deploy nodes more efficiently and precisely in the harsh underwater environment. Their adaptability and autonomy allow them to deal with specific problems like uneven terrain and water currents. They also pointed out that the combination of intelligent algorithms and robot collaboration considerably enhances UWSN coverage. For instance, the CRO algorithm has been shown to achieve an average coverage rate of 95.66%, outperforming traditional methods. Also, optimized node deployment enhances the accuracy and efficiency of environmental monitoring, which is crucial for applications like marine resource exploration and scientific research [13]. Fernandes et al. demonstrated the Reliable and Efficient Routing for Water Quality Monitoring (REWQ) mechanism, which employs the Cuckoo Search (CS) method. This strategy focuses on optimizing routing paths to provide consistent data transfer while reducing energy consumption. This technique attempts to improve data transmission reliability and energy efficiency in UWSNs by tackling typical issues, including restricted bandwidth, high energy consumption, and node mobility [14].

The rest of the current paper was planned as follows: Section 2 shows the problem’s preliminary stages, details, proposal model, and suggested algorithms. Section 3 presents the simulation results and evaluations of the proposed model.

## 4. Proposed Deep Filtration Learning Model

The word “deep” in deep learning refers to the network’s utilization of many different layers. The deep learning approach used in this work is a multi-layer neural network with a deep hierarchy that depends on the clustering method in distribution sensors underwater. This enables nodes to learn filtration principles based on large amounts of data. Hence, the levels of designated sensor neural networks attempt to simulate human brain behaviour—matching Qualitative Liquidation (QL) in implementing the capacity of inventorying and sorting traffic data, whether anomalous or accurate. While the proposed UWSN neural network has two hidden layers, the first layer can produce approximate predictions, and the second layer can assist the first layer in optimizing prediction and refining for accuracy. The cluster head (CH) node supervises the proposed deep filtering–learning approach.

### 4.1. Problem Definition

In the suggested deep filtering–learning model scenario, the UWSN consists of numerous clusters governed by a fusion centre. The fusion centre collects all data obtained from network devices and transmits it to the back-end system. It connected the intended underwater wireless sensor network to positioning systems via IoT within various ponds in fish farms. Farming sensors were designed to meet the International Resource Identifiers System (IRIS) criteria and were powered by a communication M64a transceiver manufactured by Water Linked AS (Trondheim, Norway). An ESP8266-01 Wi-Fi module manufactured by Espressif Systems (Shanghai, China). And the Underwater GPS G2 positioning system manufactured by Topcon Positioning Systems (Livermore, CA, USA). In addition, these sensors are distributed in nine clusters throughout one pond. Within a WSN, the cluster consists of one head node and five sensor nodes, which are spread randomly. Furthermore, each sensor node in the cluster has an initial power source, a processor unit, a memory, an RF transceiver, and sensor devices. The sensor nodes’ primary transmission mode is one-to-many broadcast. The transmission range of each sensor node is fixed. The sensor node saves the sensed data locally in its memory before sending it to its neighbours and the cluster head.

In the proposed method, all cluster nodes have the same sensing radius and are aware of the distances between them and the fusion centre. The proposed method is based on the genetic algorithm (GA) in the clustering method, which is used to establish the optimal network structure that minimizes energy exhaustion after each transmission round. During the optimization phase, each GA chromosome serves as a cluster head identification map. A gene in a chromosome determines whether the relevant node serves as a cluster head. Given a cluster head, node clusters are generated using the closest neighbour criterion, and the fitness of a chromosomal WSN structure is calculated by evaluating all clusters. This method greatly extends the network lifetime by balancing the energy consumption among all sensor nodes in the WSN. In general, the UWSN experiences substantial communication problems as a result of its short lifespan, which is caused by a variety of issues such as insufficient communication overhead and sensor malfunctions during deployment operations due to harsh environments. Defects in data dissemination, caused by missing and noisy data, result in network misbehaviour and incredibility. In this paper, a predictive learning model is developed to address the deployment issues and energy challenges in the designated network. The suggested methodology considerably reduces the pace of data transmission for the UWSN by forecasting future data and missing measurements and then reducing the noise associated with the sent signals, improving energy efficiency. The following subsection demonstrates a description of the employed network architecture.

### 4.2. Network Architecture

The proposed designated network architecture was designed as follows:

- Input Layer: Data or sensory readings from dispersed sensors in the ponds are received by this layer.
- Layers that are Hidden: These layers extract features. They are made up of neurons that alter the incoming info. One kind of RNN, or recurrent neural network, that can preserve long-term dependencies in sequential data is the LSTM or long short-term memory. Typical hidden layer kinds are as follows:
  1. Convolutional Layers: These provide input data to convolutional neural networks (CNNs). Long short-term memory (LSTM) is a type of recurrent neural network (RNN) that was created to address the issue of disappearing gradients that conventional RNNs encounter. Because of this, LSTMs work especially well for jobs involving time series and sequential data. They keep track of earlier inputs.
  2. Fully Connected Layers: These are used in both CNNs and RNNs. Every neuron in this layer is connected to every other neuron in the layer before it.
- The layer of Output: This layer generates the last forecast. It frequently generates its products from the following one LSTM cell and five gates:
  (a) Memory Cell: The memory cell in LSTMs is capable of storing data for extended periods.
  (b) Three gates are used by them to regulate the information flow:
    1. The input gate selects what fresh data should be stored.
    2. Forget Gate: This gate selects which data to ignore.
    3. Output Gate: This gate regulates what data are sent out to capture spatial hierarchies, and they use convolution processes.

### 4.3. Model Description

The accuracy of deep learning algorithms has improved as more datasets are added. However, deep learning has limitations; it may not perform well with small test data, and altering the definition results can be difficult. The LSTM deep learning algorithm was followed in the proposed implementation for cancelling the noise accompaniment to the emitted signals. Long short-term memory (LSTM) adjusts and fits itself for accuracy using gradient descent and backpropagation underwater. The proposed approach uses LSTM to produce more precise predictions regarding turbidity and pollution in fish farm monitoring using hidden layers. The LSTM algorithm was trained and supervised on a series of training sequences for clustering network elements. Supervised learning uses labelled datasets to categorize and forecast. At this phase, they will correctly diagnose data readings. The suggested network’s output layer supported the CHs nodes. These are based on deep predictive learning, in which a model learns to become more accurate in completing an action in an environment involving lost data.

A. LSTM Filtration Algorithm for Proposed Network Learning

Long short-term memory (LSTM) is a recurrent neural network (RNN) architecture that is commonly utilized in deep learning. It excels at capturing long-term dependencies, making it an excellent candidate for sequence prediction problems. LSTM, unlike standard neural networks, contains feedback connections that allow it to interpret complete data sequences rather than just individual data points. As a result, it is extremely successful in understanding and predicting patterns in time series.

**Figure 1:**
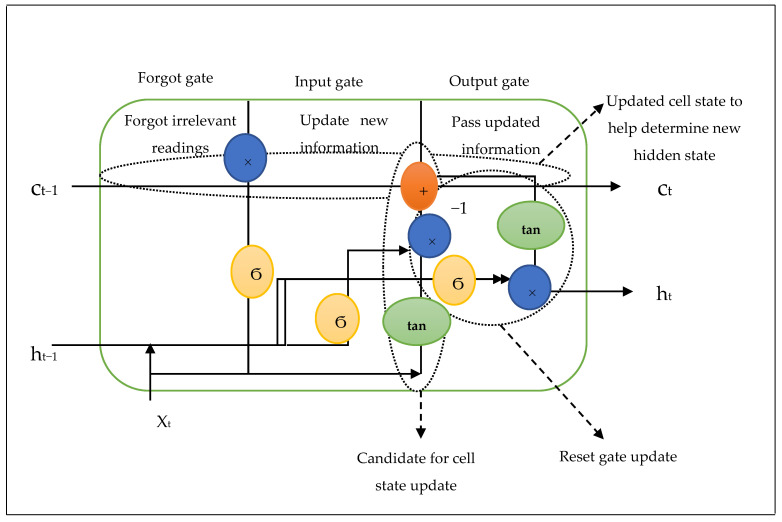
The suggested LSTM architecture for the proposed UWSN.

The LSTM was implemented at the cluster head level in this work. The LSTM design was divided into three phases, each of them performing a specific purpose. In the first phase, after the clustering sensors communicate their readings to the CH, the CH begins to sort the significance of the produced signals to determine if the sensor sender’s previous timestamp should be recalled or forgotten. When a sensor disconnects from the clustered connection due to battery failure (dead node), the CH begins to clean its brain and forgets the prior information. In the event of a connection, the cell prepares all active sensor readings and then saves them in its brain to be remembered later if necessary. The cell strives to learn new information from the input in the second phase. Finally, in the third phase, the cell changes the information from the current timestamp to the next timestamp. This cycle is regarded as a one-time phase. The LSTM unit embedded in the CH has three gates that control the flow of data into and out of the memory cell. There are three sorts of gates: forget, input, and output, as well as a memory cell. A memory cell is equivalent to a layer of neurons, and the data flow of the designated network is directed by feed-forward neural networks. Every neuron has a hidden layer as well as a current state. The prior timestamp’s hidden state is h (t − 1), whereas the present timestamp’s hidden state is ht. The information, as well as all the timestamps, are carried by the cell state. Figure 1 shows that the LSTM has cell states for prior and current timestamps, denoted by c(t − 1) and c(t), respectively. 

The suggested paradigm allows the head node to incorporate two filters at the level of the LSTM operation. The first one is the adaptive Cuckoo Filter (ACF), and the Kalman filter is the second. The ACF assesses whether an emitter node belongs to a set. The Kalman filter optimizes the estimation of state values in a dynamic underwater sensor networking system. The LSTM feeds the head node to complete the two jobs. Once the first task is accomplished, the transmitted readings will belong to the clustering elements; and the head node should realize it is no longer employing the ACF filter. The forget gate allows the head node to forget about the initial task. In an LSTM cell, the initial step is to decide whether to keep or discard data from the previous time epoch. The forget gate keeps a hidden state that serves as memory and is refreshed at each time step with the input data and the previous hidden state. The following are the roles that these gates played in the LSTM design.

- **Forget gate**: The input readings (*x_t_*) are coupled with the previous outputs to form a fraction between 0 and 1, which defines how much of the previous state must be preserved (or, how much of the state should be forgotten) at the forget gate. Equation (1) is as follows:
  (1) $$f_{t}=\partial (x_{t}\times y_{t-1}\times u_{f}+h_{t-1}\times w_{f})$$
  where
  If  f=1, *C*_*t*−1_ × *f_t_* = *C*_*t*−1_ means the cell remembers prior readings and uses the values of scales in the prediction.
  If  f=0, *C*_*t*−1_ × *f_t_ = 0*; the cell forgets everything and prepares itself to accept new readings.
- **Input gate**: The input gate operates on the same impulses as the forget gate, but its goal is to determine which important information should be added to the cell state and hence gain long-term memory. A hyperbolic tangent activation layer was used to alter the obtained input values by manipulating a hidden state at timestamp (*t* − 1) and input × at timestamp (*t*). Then, it decides which values are updated in preparation for accessing the later gate to obtain the appropriate output values. Using Equation (2), the product of the new information will be between −1 and 1.

(2) $$i_{t}=tanh\left(w_{i}\times x_{t}+w_{h}\times h_{t-1}+b_{k}\right)\;$$

If the value of *i_t_* is negative, the information is deducted from the cell state. Otherwise, the information is added to the cell state at the current timestamp. The ACF filter was integrated into the input gate to filter input data by reacting to false positives and deleting them for subsequent manipulations. Following the occurrence of new information, there will be a test result intended to arrive at inaccurate signals, indicating the presence of a specific condition or attribute. A false positive test was performed using a diagnostic instrument meant to determine the cluster affiliation. It was used to identify the source of a substantial number of false positives in the sensor and its affiliation with any cluster. The proposed method was produced to suit a considerable flow of sensing data under sensor monitoring, which may frequently correlate with flow identifiers. As a result, a theory to search for false positives is used, followed by repeated searches for each sensor in each cluster. By removing false positives, the false positive rate can be significantly reduced. The ACF filter stores the replies of failed sensors in a hash table. To optimize the efficiency of the cluster heads, it avoided sensor readings that fluctuated responses to false positives via varying epochs. It is worth mentioning that the coming value from the failed sensor will not be directly incorporated into the cell state. The cell state ($N_{t}$) was computed as follows:

(3) $$N_{t}=f_{t}\times c_{t-1}+i_{t}(1-e^{-\mu }{)}^{k}\;e^{-\mu }$$

- **Output gate**: During this gate, processing the results of the input gate must be finished. When the output gate receives the input, the prior state is gated again to generate another scaling fraction, which is combined with the output of the function block (*tanh)* to produce the current state. A Kalman filter is then applied to this yield product. The output product and the state are then passed back into the Kalman filter implanted in the LSTM block to gain the estimated state from the previous time step. No preceding set of observations and/or estimates is required here, and just the current measurements are required to generate a pure estimation for the current state. In contrast to data batch estimation, it employs a series of measurements taken over time, including statistical noise and other inaccuracy values, to produce estimates of the unknown variables that are more accurate than those based solely on a single measurement. As a result, the filtered output signal was expected to be free of noise. The converged output (${\hat{y}}_{k})$ is represented by the following output gate’s equation:
  (4) $$y_{k}=\delta (f_{t}\times x_{t-1}+b_{k}\times u_{k})+{h_{t-1}\times w}_{k}+Q_{k}$$

It added the *sigmoid* function, ranging between 0 and 1, and the *P_k_* and *tanh* functions in the calculations to infer the updated cell state to determine the current hidden state, as illustrated below.

(5) $$P=f_{k}\times y_{k}u_{k}+Q_{k}$$

Then,

(6) $${\hat{P}}_{k}=(i_{k}-k\;x_{k}\;\times h_{k}\;)\;P_{k}$$

Hence,

(7) $${\hat{y}}_{k}={\hat{P}}_{k}-h_{k}x_{k}$$

where *x_t_* is the current time epoch’s reading; *y_t_* is the coupled previous outputs to generate the predicted signals; *u_(f_*,*_k)_* is the related weight with the sensor reading; *h_t−1_* is the previous time step’s hidden state; *w_f_* is the weight of the hidden state in the forgetting step; *w_i_* is the weight of the input reading for the input state; *w_(h,k)_* is the input weight matrix connected with the hidden state in the input step; *h*_*t*−1_ is a concealed state at the preceding timestamp; *b_k_* is a control-input model bias factor that is applied to the control vector u_k_; *C*_*t*−1_ is the cell state at the current timestamp; $e^{-\mu }$ is the approximation for the likelihood that there are no false positives in a certain hash table; $N_{t}$ is the updating cell state at the current timestamp; *w_k_* represents the process noise, which is supposed to come from a zero mean multivariate normal distribution with covariance (*Q_k_*); Q*_k_* is the coefficient of the signal noise covariance; ${\hat{y}}_{k}$ is the convergence value of the predicted signal; $P_{k}$ is the updated convergence estimate of the a posteriori signal; *h_k_* is the concealed state at the current timestamp.

B. Parameter Settings

The deploying processes handled the following parameter settings:

(a) Learning Rate: Establishes the amount by which the model’s weights are updated during training. The value of 0.001 is a typical starting point.
(b) Batch Size: The number of training examples used in one iteration. Typical values range from 32 to 256.
(c) Number of Epochs: The number of times the entire training dataset passes through the network. This can range from 10 to 100 or more, depending on the problem.
(d) Filter Size: The dimensions of the convolutional filters, commonly 3 × 3 or 5 × 5.
(e) Stride: The step size with which the filter moves across the input. A stride of 1 means the filter moves one pixel at a time.
(f) Padding: Adding zeros around the input to preserve spatial dimensions after convolution. The used options are: “valid” (no padding) and “same” (padding to retain dimensions).

The next subsection describes the self-filtration phase conducted by cluster sensors for optimizing the efficiency of the designated network. Algorithm 1 illustrates how adjusting the weights minimizes the error function.

C. Self-Filtration for Sensors

The purpose of self-filtration for sensors is to produce several benefits. First, it improves the accuracy of sensor data. Second, it reduces the amount of data that needs to be transmitted to the CH. The performance of the filtration mode was accomplished in the sensing session. It is adequately correlated with the generation mode of the readings.

1. The sensor (Si) aggregates the surrounding data throughout different epochs without making predictions. This aggregation process can be completed using a median filtering method.
2. After aggregation, the aggregated data, in every Si in the cluster, are transferred to the integrated FIR filter and ALE filter, which are installed in the sensing slice in the sensor circuit.
3. A proper estimation of the aggregated data is made through this mode. The estimation process was completed using deep learning by the CH in each cluster.
4. If the Si does not receive any data during one of the epochs, it will transmit a *Break* flag to the CH, which includes the ID node and the time. This break flag indicates to the CH that the Si is unable to deliver data for that time.
5. In this session, the CH will use the prediction method described in Equations (1)–(7), which express the prediction process used to approximate the data for *S_i_* for the missing period.

In this paper, the sensor’s performance improved by combining the FIR and ALE filters. An FIR filter is a non-recursive filter that has a limited impulse response. This means that the filter’s output is only affected by the current and previous inputs, and not by any past outputs. As a result, FIR filters are essentially stable, and this gives them a substantial advantage over infinite impulse response filters. Further, we attempted to incorporate adaptive filters into the FIR filter for signal separation by employing a structure known as an adaptive line enhancer (ALE). It uses the integrated filter to properly separate the signals and gain the pure signal for adaptive line enhancement of the recorded underwater signals by estimating a measured signal *x*(*n*), including two signals, an unknown signal of interest *v*(*n*), and a nearly periodic noise signal *p*(*n*). The purpose is to remove the noise signal from the measured signal to retrieve the signal of interest, as well as to amplify the frequencies, filter out undesired signals, and obtain equalizing signals. The FIR filter was used to filter and amplify the signal received by sensor S_i_. This was completed by multiplying each sample of the input signal by a corresponding filter coefficient and then adding the results to the ALE adaptive filter to produce the output signal. The filter coefficients influence the integrated filter’s frequency response and the hesitancies’ recurrence, accompanied by the original signal over a particular period or in a given sample. The hesitancies are the rates at which a vibration occurs that constitutes a wave, usually measured per second. This could be completed to increase the signal’s signal-to-noise ratio (SNR). The downstream circuitry was processed, through:

1. Set up the ALE filter. This entails determining the signal delay D and the filter length L.
2. Set the integrated adaptive filter coefficients to their default values.
3. Repeat the measurement process.
4. Complete the following for each sample of the sensed signal:
  i. Compute the adaptive filter output; the process involves separating the unadulterated input signal (${\overset{⃑}{u}}_{k}$), multiplying the estimated input signal by the adaptive filter coefficient and the weighted vector, and then subtracting the values of undesired signals from the resulting signal.
    (8) $${\overset{⃑}{u}}_{k}(n)=2\;\mu x_{k}\times w_{k}-∐v\left(n\right)p(n)$$
    (9) $${\hat{y}}_{k}(n)=\sum_{i=0}^{n-1}w_{k}{\overset{⃑}{u}}_{k-i}$$
  ii. Determine the error signal. This is the difference between the measured signal and the output of the adaptive filter.
    (10) $$e_{k}(n)={\overset{⃑}{u}}_{k}(n)-{\hat{y}}_{k}(n)$$
  iii. Update the weights vector with the adaptive filter coefficients. This was accomplished through the use of the proposed adaptive filtration learning method. The adaptive filtering–learning algorithm adjusts the adaptive filter coefficients so that the mean squared error between the measured signal and the adaptive filter output is as small as possible. Table 1 lists the acronyms and notations used to denote signals. The adaptive integrated filter learns to cancel out the almost periodic noise signals over time, leaving only the signals of interest.
    (11) $${\overset{⃑}{w}}_{k}(n)=w_{k-1}+2\mu \;e_{k}{\overset{⃑}{x}}_{k}$$
    Consequently,
    (12) $$e_{k}(n)={\hat{y}}_{k}(n)-{\overset{⃑}{w}}_{k}(n)\;{\overset{⃑}{u}}_{k}(n)$$
  iv. Assign the discrete signal. This necessitates *X* (*N*^2^) complex multiplications and additions, where *N* is the signal length. To circumvent this computational cost, the computational backdrop of the FIR filter is used, which minimizes the computational complexity of *X*(*N*^2^) complex multiplications and additions, as follows:
    (13) $$X_{k}=\sum_{n=0}^{N-1}e^{2\mu k(\frac{n}{N})}\;{\pi x}_{k}$$

*X*(*N*^2^) is transformed into an array with a dimensional vector of indices *n* = (*n*_1_,*n*_2_, …*n_d_*) by a collection of *d* nested summations, *n_j_* = *0*, …, *N_j_*_−1_, for each *j*, where the division *n*/*N* is performed element-wise. The yield (*x_k_*) is a sequence of sets of one-dimensional discrete signals performed along one dimension at a time (in any order).

D. Training Process

The training process included in sequences of the steps are as follows:

(a) Data Preparation: Collect and preprocess the data, and split the dataset into training, validation, and test sets. Normalize the data if necessary.
(b) Initialization: Initialize the network’s weights, according to the above subsection.
(c) Forward Propagation: Pass the input data through the network to obtain predictions.
(d) Error Calculation: Compute the loss using a suitable error function (Algorithm 1—Step 11), for classification tasks.
(e) Backward Propagation: Calculate the creditable membership of the clustering members concerning each weight using the propagation function. The propagation function of the feedback filters f(z) becomes the following:

(14) $$f\left(z\right)=\frac{Y(z)}{x(z)}=\frac{\sum_{i=0}^{P}b_{i}z^{-i}x\left(z\right)}{\sum_{i=0}^{Q}a_{j}z^{-j}y\left(z\right)}$$

(f) Weight Update: Update the weights using an adaptive filtering–learning algorithm, iteratively, for testing the model on the test set to evaluate its performance (Algorithm 1—Step 8).
(g) Iteration: Repeat the forward and backward propagation steps for a set number of epochs or until the model converges.

**Algorithm 1:** The adaptive filtering–learning algorithm.**Input**: a set of sensors within the cluster (N_cluster_), *X*(*n*) = [*x*(*n* − *1*), *x*(*n* − 2), …, *x*(*n* − *K +* 1)], 
*u_k_*(*n*) = desired response at time *n*, *n* = 0, 1, 2, …, *n* + 1 
**Output**: predicted value ${\hat{y}}_{k}(n)$ and *error e_k_* (*n*) 
**Initialization**: *w_k_*(*n*)  Step 1*: **for each** n* = *I in N_cluster_*
 Step 2: $w(n)=Ŵ(n-1)+\frac{\mu }{\gamma +x\left(n\right)}e\left(n\right)x(n)$
 Step 3: ***for***
*i* = *1 to k do*
 Step 4: ~${\hat{y}}_{k}(n)=\sum_{i=0}^{n-1}w_{k}{\overset{⃑}{u}}_{k-i}$
 Step 5: $e_{k}(n)={\overset{⃑}{u}}_{k}(n)-{\hat{y}}_{k}(n)$
 Step 6: ***end for***
 Step 7: ***for***
*j* = 1 *to M do*
 Step 8: ${\overset{⃑}{w}}_{k}(n)=w_{k-1}+2\mu \;e_{k}{\overset{⃑}{x}}_{k}$
 Step 9: ***end for***
 Step 10:$\overset{⃑}{\;y}\left(n\right)=\left[1-\mu \right]w\left(n\right)+\mu e\left(n\right)x(n)$   Step 11: ~ $e_{k}(n)=\overset{⃑}{y}\left(n\right)-{\overset{⃑}{w}}_{k}(n)\;{\overset{⃑}{u}}_{k}(n)$
 Step 12: ***end for each***
 Step 13: ε(z) = y(z) − *ʎ* ${\overset{⃑}{w}}_{k}\;X(Z)$   Step 14: ***Return***
$\overset{⃑}{\;y}\left(n\right)$, e(n)

## 5. Simulation and Discussion

Monitoring aquatic environmental factors in real time is crucial. So, underwater wireless sensor network integration with positioning systems under IoT technology has the potential to transform the real-time monitoring of underwater ecosystems. In the implementation of the proposed network assumptions, the International Resource Identifiers System (IRIS) requirements supported by the communication modem M64a transceiver, ESP8266-01 Wi-Fi module, and positioning system Underwater GPS G2 (Global Position System Generation 2) were used. The M64a transceiver is designed to operate in low-power, low-bitrate, and long-range modes. M64a provides a dependable acoustic communication link between two subsea locations with limited space, weight, or power. Data are exchanged at 64 bps across a robust two-way half-duplex acoustic link that is employed to assure data transfer reliability, and the GPS G2 gives a single locator with reliable, accurate, and robust acoustic locating. To execute the proposed network assumptions, the constructed network involved (*N*) sensor nodes distributed in clusters. Each cluster in the manufactured network uses GA to select the head node. In the evolutionary algorithm, each node in the cluster sends enclosed signals about its energy and geographic region to a hub. The planned network has (N) sensor nodes that are distributed in clusters. Each cluster in the manufactured network uses GA to select the head node.

In every deploying round, the elected node receives a hello message from the base station, which allows it to declare itself as a cluster head (CH) to the other cluster members. In the algorithm’s implementation, any sensor node can choose to be a cluster head if it has not received an announcement message from the head. In this situation, this node sends out an announcement about the head selection to the cluster nodes to promote itself as the group leader. Cluster members are sent a join message to the node providing the announcement, which announces their connection with the cluster. Each GA chromosome serves as a designating map for the cluster leaders. A gene on a chromosome controls whether the appropriate node appears as the group’s leader. Cluster members are sent a join message to the node providing the announcement, which announces their connection with the cluster. Each GA chromosome serves as a designating map for the cluster leaders. A gene in a chromosome controls whether the relevant node appears as the group’s leader. The closest neighbour rule is then used to build the cluster’s nodes, and the importance of a chromosomal WSN structure is assessed by analyzing all groups. During the steady-state phase, the nodes in each cluster can begin sensing information and communicating it to their cluster head for the predetermined transmission time. Because the cluster head (CH) is typically located a long distance from the base station (BS), communicating with it requires a significant amount of energy; therefore, the head must be elected each cycle. Following direct data sensing, each sensor node broadcasts the individual data to surrounding nodes as well as the cluster head across a specified period. Implementing sleep modes for sensors can significantly reduce energy consumption. In each cycle, every node in the cluster determines the energy consumption using the following equation:

(15) $$E_{OVERALL}=E_{I}–\sum E_{IDLE},E_{SLEEP},E_{TRANS}E_{REC}$$

Table 2 displays the simulation results for embedded underwater sensors. As a feature, we selected pH (the potential of hydrogen), Ammonia (NH_3_), temperature, turbidity, and fish as the goal attributes. The practical results are divided into two sections, sensory filtration and prediction learning. In our investigation, we used pH, temperature, and turbidity as aquatic environment factors. It created an IoT platform for sensor-based real-time aquatic environment monitoring. Through monitoring the water quality of five ponds, the sensors recorded four attributes: pH, Ammonia (NH_3_), temperature, turbidity, and fish. The studied dataset included 12 columns and 4591 rows. In this study, fish is the dependent variable, and the others are the independent ones. There are 11 different fish categories, 86 different pH values, up to 102 different NH_3_ values, 46 different temperature values, and 85 different turbidity values. The proposed system is designed as an embedded system with sensors and an alarm coupled with a GPS application. A variety of sensors are utilized to measure parameters such as the pH, temperature, turbidity, and fish taxonomic group. These sensors are linked to a microcontroller board, which also includes an alarm system. The sensors collect data from the water and transfer them to the cluster head (CH) via the digital signal microcontroller (DSP) for processing and storage in the related memory. The proposed filtering–learning system for sensor nodes in the designated underwater network was created in MATLAB (https://ww2.mathworks.cn/products/matlab.html). The data are employed during deployments for two types of scenarios during deployment rounds. The initial test was conducted on the fish farm’s aquatic data, which were obtained from the University of Dhaka in Bangladesh. The second scenario of inspection carried out real trials at one of our government fish farms. To validate the proposed model, data was collected from 270 sensors laid in five ponds of different sizes and settings. Each pond included 54 sensors distributed in a decentralized manner. The designated network was constructed using a clustering method in which a genetic algorithm (GA) was employed to determine the ideal structure of the designed network after each transfer cycle, based on a periodic reevaluation of its energy. During the optimization procedure, each chromosomal GA represents a map of the cluster head identification. A gene on a chromosome identifies the appropriate node, whether the cluster head or the ordinary node is utilized. The head node is then built based on the closest neighbour rule, and the ability of a chromosome-driven WSN structure is established by all cluster evaluations. The accuracy, the True Positive Alert (TP) rate, and the amount of noise cancellation achieved for the transferred data were measured.

A. Sensory Filtration for Aquatic Monitoring

The datasets were observed in real-time monitoring and control systems for fish farming. Each fish pond is a sensory cluster in the wireless sensor network managed by CH filters. It is an embedded microcontroller designed with integrated filters (FIR and ALE) and a Global Positioning System (GPS), and it is linked to many sensors via a wireless communication module. The aquatic data such as the water surface temperature, air temperature, pH, NH_3_, water bottom temperature, and turbidity are measured in real-time by having numerous sensors with homogeneous constructions. Each sensor, in the pond, measures one of the specified characteristics, and the date and time of measurement are also recorded. The data are collected by the sensors and then transferred to the cluster head, as stated in Table 3. These data were recorded in the CH’s cluster memory and recovered for the real-time analysis and forecasting of the missing data readings.

In this mode, the dataset was filtered using a resampling option to observe the present relationship between the dataset’s instances and characteristics. Also, four sensors were employed in the filtering process to collect real-time data from each pond’s water. The sensors are a pH sensor, a bottom temperature sensor, a turbidity sensor, and an Ammonia sensor. Each sensor begins by determining the monitored value (*xi*), which is regarded as a noisy signal. As a result, the filtering integration phase works to refine *xi* by measuring the signal formed by the sum of these two signals, an unknown signal of interest *v*(*n*), and a nearly periodic noise signal *p*(*n*). This signal is known as *x*(*n*), and it is treated using two integrated filtering processes. The signal *x*(*n*) is first entered into an adaptive line enhancer (ALE), which is based on the simple principle of linear prediction. A nearly periodic signal can be completely predicted using the linear mixtures of its prior data samples. In this phase, any delayed data of the measured signal *x*(*n*-*D*) are entered as the adaptable filter’s reference input signal *x*(*n*), and the desired response signal *d*(*n*) is set equal to *x*(*n*).

**Figure 2:**
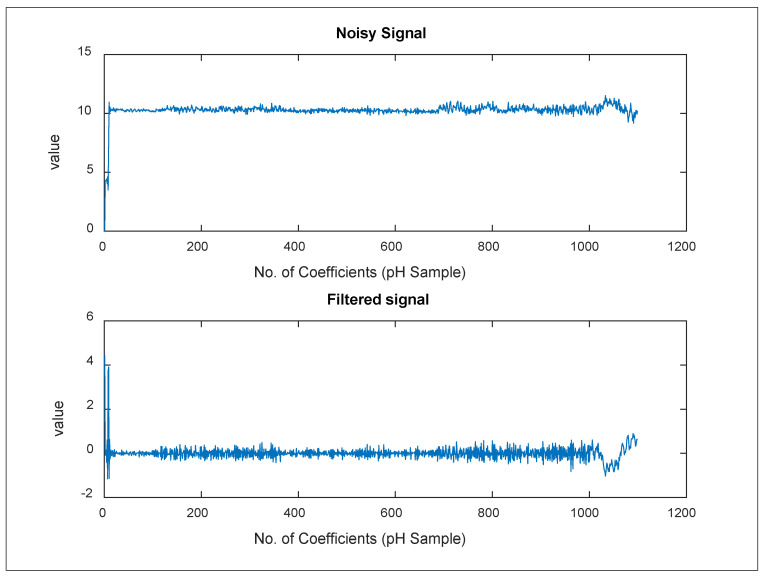
An illustration of eliminating the noisy value of the pH sample through filtration experiments.

Table 3 displays a sample of noisy signals processed as input data *x*(*n*) to the integrated filter in this manner. The parameters used through applying the proposed system were the signal delay length *D* and the filter length *L* that were employed in the adaptive linear estimate. The duration length of the delay is proportional to the degree of correlation in the signal of interest. So, we simply chose a value of *D* = 100, which was then modified in subsequent rounds. In addition, *L* = 32 was used for the adaptive filter. Following that, we used certain block adaptive algorithms that need the vector lengths for *x*(*n*) and *d*(*n*) to be integer multiples of the block length. We set *N* = 49 as the block length. The output signal y(n) comprises a considerable portion of the periodic sinusoid, whereas the error signal *e(n)* contains unknown information. We might plot *e*(*n*) vs. *v*(*n*) alongside the residual signal *e*(*n*) − *v*(*n*) in the same plot, shown in Figure 2. It is worth noting that the system converged the signal after approximately 3 s of adaptation with this step size by subtracting a pure sinusoid from the original signal’s sinusoid, as shown in Table 4.

**Figure 3:**
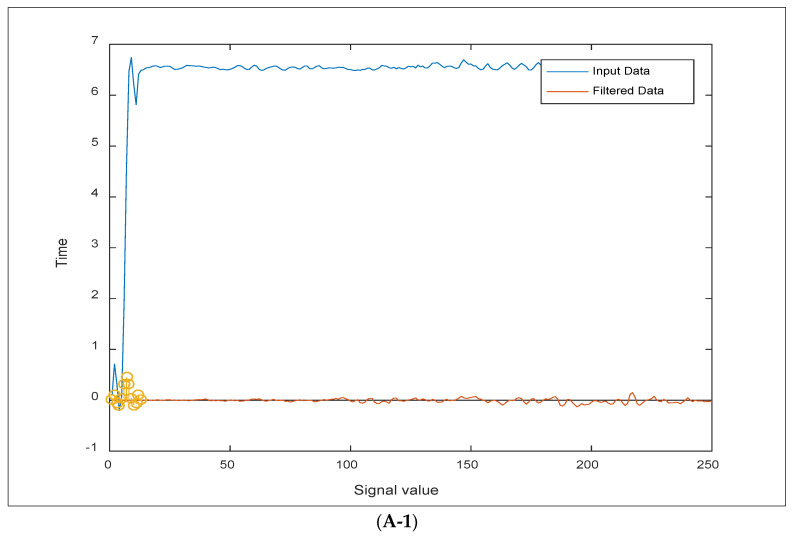

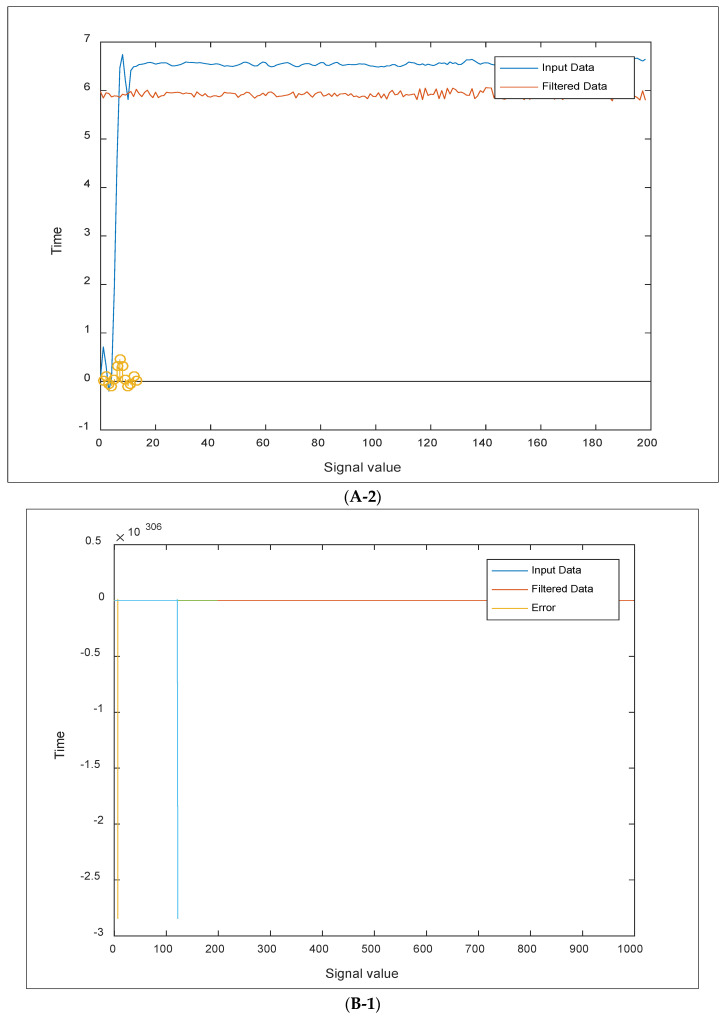

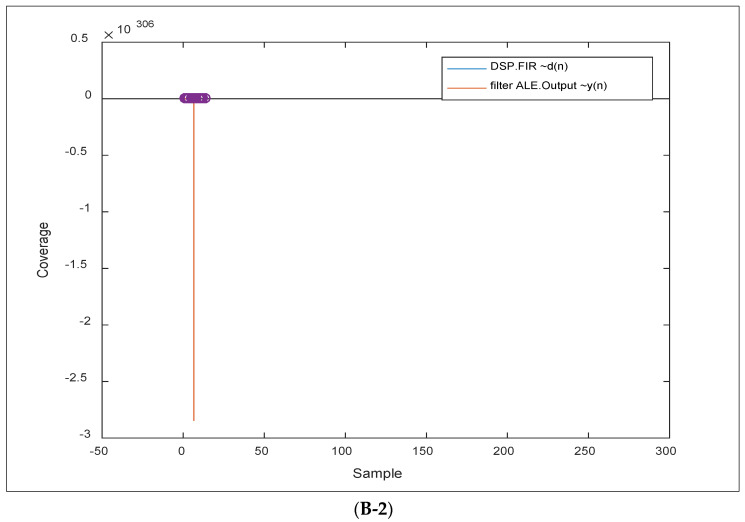
Results of an underwater sensor regraded with (**A-1**,**A-2**) free noisy signals of filtered samples of NH3; whereas (**B-1**,**B-2**) illustrate a zero error rate and the convergence of real-time and filtered samples of turbidity values in some ponds.

Table 4 summarizes the yields of an integrated filter in the sensory mode via a treated pH sample. It was demonstrated that the size of the noise was finally eliminated using the two processes of FIR filtration and ALE adaptive refining. This was accomplished by first establishing a DSP.FIR filter object that represented the filtration system to be identified. The filter coefficients were then designed using the “fircband” function. The developed filter is a low-pass filter with a stopband ripple of 0.2. After that, the signal *x*(*k*) was sent to the FIR filter. The desired signal *d*(*k*) is generated by the FIR filter as *d* = step (DSP.FIR filter, *x*). Following that, preparing the adaptive filter object necessitates the use of starting values for estimating the filter coefficients as well as the ALE step size for estimating the filter coefficients. Then, set the dsp.ALE filter’s “Initial Conditions” property to the desired initial filter weight values. For the step size, 0.8 is a fair compromise between being large enough to converge well within 250 iterations (1000 input sample points) and being tiny enough to get an accurate estimate of the FIR filter’s outputs. As a result, we discovered that the error ratio in the shown pH sample is hidden and close to zero in many samples, such as e (5.97334400000000). Table 4 also recorded convergences between real-time values and refined yields of the integrated filter, where the desired signal for the observed reading (*x*(*k*)) in the first row is 5.0980800812507553 and the adaptive signal yield (*y*) is 5.00000000000000, as shown in Figure 3.

Figure 2 shows that the suggested adaptive filter technique succeeded in managing the carry signals emitted by the studied UWNS and efficiently removed the accompaniment noise, as indicated in Table 3. Further, the adaptive incorporated filter, the FIR filter with the ALE filter, conducted periodically adaptive digital signal processing (DSP) of the emitted signals, for gaining the soft and pure signal. The inherent function of the integrated filter was developed to establish an integrated system adapted to sensor-sensed readings under harsh aquatic circumstances. Practically, the first is based on setting the filter; numerator = “fircband”, within the integrated filter used as the initial filtering procedure. Second, the yields of the ALE filter are used as relevant input data, the results of the filtering system throughout the adaption process, known as the desired signal *y*(*k*). Third, the error signal was calculated between the adaptive FIR.dsp filter’s output (*d*(*k*)) and the adaptation system’s output, ALE.dsp, denoted as *y*(*k*). The coefficients of both filters are practically identical. The error signal is minimized by multiplying μ by the sample size.

**Figure 4:**
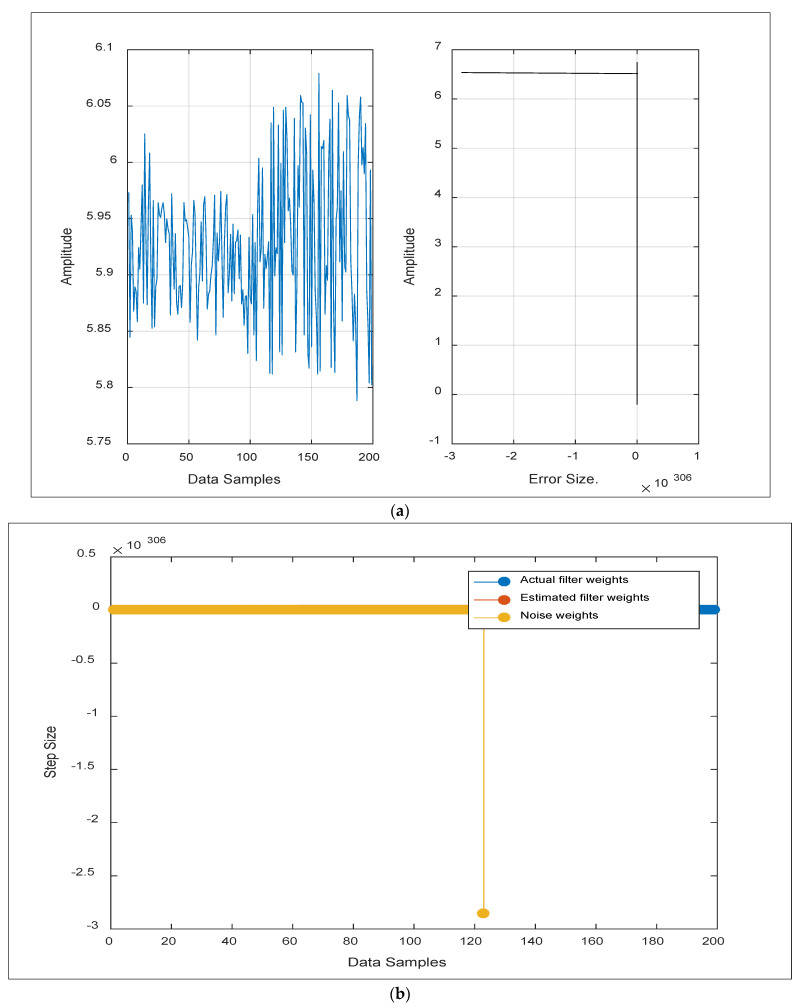
An illustration of the monitored error magnitude through integrated FIR and ALE, under the (**a**) amplitude and (**b**) step size of variant data samples.

Figure 3 depicts the reactions of a variety of incoming signal values for the aforementioned parameters in the suggested pond, which were analyzed programmatically using the integrated filter throughout numerous deployment rounds. Plotting the findings of Figure 3 emphasizes that, once *x* passes through the integrated filter, the noisy input signal (*x*) and the target signal (*d*) are identical in signalling. This was performed by launching the adaptive filter to activate the filtering system, which works in tandem with the ALE filter to produce an adaptive refined signal (*y*). The adaptive filter’s output (*y*) is the signal that has converged to the intended signal (*d*), minimizing the error (*e*) between the two signals to a small value for the product signals. As seen in graphs (3-A and 3-B), the output signal matches the target signal, rendering the difference between the two negligible as the error magnitude approaches zero. Figure 4 below shows the error magnitude through the integrated FIR and ALE.

Figure 4 shows that the weights vector (*w*) has a significant impact on the coefficients of the integrated filters, especially used to start the suggested system (FIR filter). To confirm convergence, we calculated the numerator of the FIR filter and the estimated weights of the adaptive filter in the first process’s step size. We increased the step size to 0.2 to account for varying signal amplitudes. It was discovered that the estimated filter weights closely match the actual filter weights. As a result, the weighting size of the noise-accompanied signal was reduced to zero, corroborating the results reported in the previous signal plot.

B. Distributed Prediction Learning

**Figure 5:**
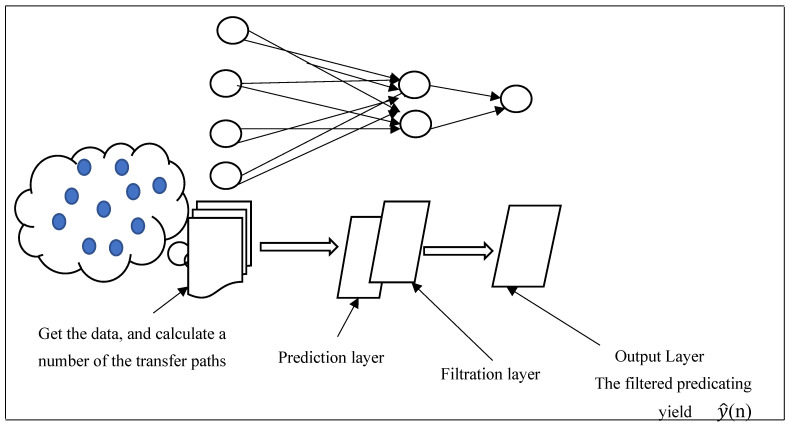
A succession of the implementation technique demonstrates the proposed mechanism of distributed prediction learning.

A distributed method for deep learning in underwater wireless sensor networks was proposed in this mode. By training the middle layer as a deep learning block, the integrated filter (ACF) and Kalman filter were embedded into it. Then it was put on the CH for each cluster. This was to perform distributed data processing to remove the noise accompanied by outgoing signals and achieve prediction for lost signals. In this manner, the suggestion is a distributed deep learning application at the cluster head level in each deploying group in underwater wireless sensor networks; see Figure 5.

**Figure 6:**
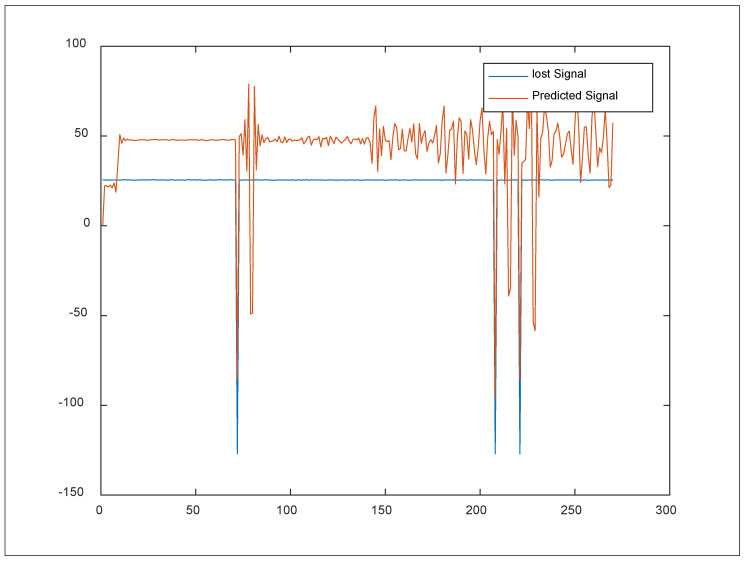
The predictive procedure of the filtered LSTM neural nodes for temperature values in iteration 60.

Figure 5 depicts the operating procedure phases for the suggested method. First, the input layer is assigned the first multiple layers as an input port for coming sensor readings. Then, an intermediate layer is assigned to the CH that receives the data and calculates the number of transfer paths throughout the round. Second, the remaining intermediate layers are divided into two sub-layers and assigned to the alerting notifications and predicted yields for relay nodes in each cluster, which is known as the fully connected layer or the output layer. Third, during the deployment cycles, each CH in the cluster must synchronically deliver the output result to the base station via wireless communications via IOT. Fourth, if the clustering nodes do not transport data to the hidden layer regularly across several hops, the cluster head does not receive any transfer data from these sensors. Finally, in the alerting layer, CH checks the number of multiplex notifications to diagnose the node’s status, performs prediction computational processing with the integrated adaptive filters, and then transfers the predicted product ($\hat{y}$(n)) to the base station. Fifth, the CH performs the computational processing of the remaining middle layer by training the cell with samples of the filtered products coming from the learning intermediate layer if the number of hops is sufficient; however, the data transfer was not complete upon dispatching to the CH. Figure 6 depicts the yields concluded from the prediction paradigm for a sample of the dataset simulated by the neural network. As illustrated, a perfect signal strength emerged from a sector of missing temperature data via dispersed prediction coverage employed by the network’s filtered LSTM neural nodes. This was conducted across 300 microseconds during one of the deployment epochs during iteration 60.

In Figure 6, inconsistencies in the data were widespread, as were missing or out-of-range values. There were also long uninterrupted noise segments. Therefore, the data were cleaned by replacing missing or noisy values with preceding values in time (filling the missing temperature value with the most recently recorded temperature value). We used the data readings as the current input and followed these methods to calculate the values of the three distinct gates. First, we computed the parametrized vectors for the current input and the prior hidden state for each gate using element-wise multiplication with the respective vector weights. Next, the system computed the current internal cell state. It began by calculating the element-wise multiplication vectors of the input gate and the input modulation gate. It then computed the element-wise multiplication vector of the forget gate and the prior internal cell state and summed both vectors to obtain the current internal cell state. Finally, the system determined the current hidden state by obtaining the element-wise hyperbolic tangent of the current internal cell state vector by applying $i_{t}=tanh(w_{i}\times x_{t}+w_{h}\times h_{t-1}+b_{k})$ (Equation (2)), followed by element-wise multiplication with the output gate.

The proposed method performs a check test on the final anticipated output value based on aquatic environment factors. Otherwise, the alarm system sends alert signals to the chief sensor CH about the problem position in the pond. It conducted a test to validate the final output value while taking aquatic environment aspects into account. If there is a problem with the pond, the alarm system alerts the chief sensor. Missing, unique, and distinct values of each characteristic can be examined during the noise cancellation phase. Following this method, all seven taxon groupings of fish have no missing data for any of their attributes, and the pH levels in almost all of the samples are close to four. In addition, the system forecasted the missing bottom temperature, which reached up to 30 lost readings of a variant temperature, and high-degree turbidity that did not surpass a 7.2 unique value through most of the chosen 1000 samples. It is worth mentioning that the proposed system achieved 98.5% of the sensory filtration method in most of the acquired samples and close to 99.1% of an adaptive prediction method. Compared to the recent methods, the researchers in [8,10] focused on improving water quality prediction and anomaly detection in sensory monitoring of fish farming, which is the same principle as our method. Furthermore, the wireless transceiver boards for underwater sensors deployed in genuine underwater environments are identical. Also, they measured the same water quality variables obtained from similar fish farms. The comparison methods have improved decision-making for fish farmers and reduced economic costs by minimizing labour-intensive monitoring. However, these methods were only sufficient to detect anomalies in real-time from complex time series data collected by water sensors, unless deep learning techniques are exploited to cover the issued anomalies and filter the observed sensory yields from the network to ensure the safety and sustainability of the water. Also noteworthy is their lack of limited pure signals and their reliance on sensory inputs. In addition to the misbehaviour caused by intermittent signal transmission and noisy data. The proposed method leveraged the strengths of both convolutional networks and LSTM networks in the selected comparative methods and addressed adaptive filtration for aqua signals, to efficiently identify unexpected patterns or values that could indicate water quality issues. The LSTM-CCN approach has shown impressive accuracy, with a rate of 98.5% for the sensory filtration method in the majority of the obtained data and close to 99.1% for an adaptive prediction method, while also consuming little energy during lengthy monitoring, making it highly effective in aqua environments. The suggested system achieved a detection accuracy in the monitoring field estimated at 97.8% and an error rate of ~2.3% in alerting messages throughout testing samples. The proposed method used a huge quantity of data, then processed by each node individually. Additionally, the suggested approach is more energy efficient because it reduces the amount of time that each node has to spend processing data. And each node goes into sleep mode until the next epoch of data is ready to be processed. Finally, the suggested approach is highly scalable for underwater environmental monitoring applications because it can be easily adapted to different network sizes and topologies.

## 6. Conclusions

This paper proposed a novel integrated method for predicting deep learning in UWSNs. Also, it presented a new embedded system of transceiver modules in UWSN design, and a distributed method was the behaviour of deployment in the designated network. Indeed, we conducted our experiments in government fish farms that encompassed underwater wireless sensor network integration with positioning systems under IoT. Farming sensors have specifications gained from the International Resource Identifiers System (IRIS) requirements. Furthermore, a communication modem M64a transceiver, ESP8266-01 Wi-Fi module, and positioning system Underwater GPS G2 were used. The M64a transceiver is designed to operate in low-power, low-bitrate, and long-range modes. M64a provides a dependable acoustic communication link between two subsea locations with limited space, weight, or power. However, sensors must be encased in materials that can withstand high pressure, corrosion, and biofouling. Long-lasting batteries or energy harvesting methods (e.g., from underwater currents) are essential due to the difficulty of replacing power sources underwater.

Throughout deployments, the data were collected from each sensor node in the UWSN clusters. Then, the gathered data were processed synchronically via a series of the proposed deep filtering–learning models. To improve signal emissions, cancel any interferences accompanied by the emitted signal, and gain the soft pure signal. The practical experiments revealed that water quality is heavily influenced by pH and increased water turbidity. So, the pH factor was used to determine the appropriateness of the water. Further, the main control unit calculates a variety of indicators to ensure the purity of outgoing signals that assure water quality and a favourable environment for fish growth. The proposed method enhanced the performance of a variety of sensors in challenging or unpredictable underwater circumstances. Multiple experiments on various datasets pointed to the proposed deep learning-based prediction model having high accuracy in prediction by lost events, an optimal filtering statistic for the transferred signals, and a positive alarm rate of any anomaly. However, deploying underwater sensors is a difficult undertaking that necessitates meticulous preparation and consideration of the particular obstacles presented by the underwater environment. Thus, deploying several sensors and providing redundancy can aid in data integrity and system reliability. To achieve long-term stability in UWSNs, equipment must be robust to withstand harsh underwater conditions, including high pressure, salinity, and potential biofouling; regular maintenance and checks are necessary to ensure long-term functionality and reliability. Cloud-based software can facilitate the sharing, viewing, and comparison of underwater missions, enhancing the long-term stability of data management, and regular maintenance or self-cleaning mechanisms can help prevent biofouling, which can degrade sensor performance over time.

## Figures and Tables

**Table 1:** List of parameters and their descriptions.

Parameters	Description
S_i_	Refers to the cluster’s member node.
*N*	The total number of sensor nodes observes a vector of sampled signals, which contains the current signal as well as the N − 1 previous samples. The vector of x(n) is defined as *x*(*n*) = [*x*(*n*), *x*(*n* − *1*), …, *x*(*n* − *N* + 1)]*^k^*.
*X_k_*[*n*]	The real data are represented as scalar input signals deployed by *N* sensors at time *k*, where *k* is characterized by the continuous-time signal sampling time index.
*k*	The sampling time is defined as *fs*, where *fs* is the sample frequency.
*t*	Time index: *t* = 1; 2, …,*T*.
*i*	Sensor index: *n* = 1, 2, …, *N*.
$w_{t}(n)$	The weight signal of the combination of both filters at time *t* for the short-term observation memory-based filter at instant *n*.
*u(n)*	The previous scale of an input signal.
y(*n*)	The output signal from the combined (FIR and ALE) filters.
${\hat{y}}_{k}(n)$	The predicted output (when training samples are provided feedback).
*e*(*n*)	Prediction error between the prior signal *u*(*n*) and the predicted output of both filters $\hat{y}$(*t*).
*M*	An integer representing the filter memory (the number of samples previously used).
*a_i_*	The reverse-feed filter coefficients.
*b_i_*	The feed-forward filter coefficients.
*Q*	The reverse filtering order.
*P*	The feed-forward filter order.
*X*(*Z*)	The discrete-time signal receives the input signal vector.
*Y*(*Z*)	The output signal vector is obtained by ACF filtering the discrete-time signal.
$\sim \hat{e}$(*n*)	The balance of an anticipated error signal between the training and expected samples.
*ʎ*	A factor of forgetting, where 0< *λ* ≤1 denotes the smaller value; the smaller *ʎ* helps to rule the earlier samples. Typically, ϕ is selected between 0.98 and 1.
$f\left(z\right)$	An aspect of the feedback filters’ data propagation/transfer function.
CHs	the leaders of each cluster in the assigned sensor network.
ε(z)	The absolute deviation for sensor readings from the actual observation values is provided at the cluster head (CH) as a threshold cross at time *k* by the Kalman filter.

**Table 2:** Simulation parameters of embedded sensors.

Parameter	Value
Transducer frequency	31–250 kHz
Data rate	64 bits per second
Range	200 m
Latency	1.5–2.5 s
Signal Range of GPS G2	300 m
Bandwidth	125 kHz
Communication	Wi-Fi (802.11ac/a/b/g/n)
Frequency	31.25–250 kHz (200 kHz typical)
Transmission of current draw I*_t_*	44 mA
Reception of current draw I*_r_*	10.8 mA
The current draw in sleep mode	0.2 μA.
Battery power	2AA batteries
Energy drain of GPS G2	0.375 Volts
Dispersed power	0.025 Volts
*E_I_*	Initial energy.
*E_IDLE_*	The consumed energy is in an idle state.
*E_SLEEP_*	The consumed energy in the sleep state.
*E_TRANS_*	The consumed energy.
*E_REC_*	The consumed energy in the reception.
*E_OVERALL_*	The total consumed energy.

**Table 3:** Gathered sample of readings observed from the deployed sensors for the designated fishing ponds in the proposed UWSN.

Water Surface Temp.	Air Temp.	pH	NH_3_	Water Bottom Temp.	Turbidity	Fish	Time Stamp	Water Surface Temp.	Air Temp.
25.63	22.13	5.95	7.5	31	7.2	Sing	19:11:53	25.63	22.13
25.63	22.13	5.95	7.9	25	5.6	Rui	19:01:33	25.63	22.13
25.56	22.13	5.96	8.5	32	6.0	Koi	19:02:48	25.56	22.13
25.56	22.13	5.95	8.1	29	7.2	Prawn	19:01:13	25.56	22.13
25.56	22.13	5.92	7.9	28	6.1	Sing	19:00:03	25.56	22.13
25.56	22.13	5.94	8.5	30	3.0	Tilapia	19:03:08	25.56	22.13
25.56	22.13	5.94	7.4	29	3.7	Pangas	19:06:08	25.56	22.13

**Table 4:** An overview of the pH-adapted results from the sensors in each utilized pond.

X(K)	D(K)	Y(K)	E(K)
5.97334400000000	5.0980800812507553	5.00000000000000	0.0980800812507553
5.84471900000000	5.711524107253188	2.73937801658796	−2.02785390933477
5.95300000000000	5.322718586092638	4.292949022658	−0.110615667608750
6.92412500000000	6.74139579845585	5.00895230402047	0.895230402048 × 10^−15^
6.90509400000000	6.20854989838715	6.518660090901947	0.51866009090194 × 10^−17^
5.94250000000000	5.81489146868828	5.994407024078749	0.99440702407874 × 10^−19^
6.97990600000000	6.41286461886760	5.14120845848566 × 10^22^	−0.14120845848566 × 10^−22^
6.87490600000000	6.48997667476100	5.15674236735994 × 10^24^	0.15674236735994 × 10^−24^

## Data Availability

Data are contained within the article.
